# From field to plate: How do bacterial enteric pathogens interact with ready-to-eat fruit and vegetables, causing disease outbreaks?

**DOI:** 10.1016/j.fm.2023.104389

**Published:** 2023-09-21

**Authors:** Gareth A. Thomas, Teresa Paradell Gil, Carsten T. Müller, Hilary J. Rogers, Cedric N. Berger

**Affiliations:** School of Biosciences, Cardiff University, Sir Martin Evans Building, Museum Avenue, Cardiff, CF10 3AX, UK

## Abstract

Ready-to-eat fruit and vegetables are a convenient source of nutrients and fibre for consumers, and are generally safe to eat, but are vulnerable to contamination with human enteric bacterial pathogens. Over the last decade, *Salmonella* spp., pathogenic *Escherichia coli*, and *Listeria monocytogenes* have been linked to most of the bacterial outbreaks of foodborne illness associated with fresh produce. The origins of these outbreaks have been traced to multiple sources of contamination from pre-harvest (soil, seeds, irrigation water, domestic and wild animal faecal matter) or post-harvest operations (storage, preparation and packaging). These pathogens have developed multiple processes for successful attachment, survival and colonization conferring them the ability to adapt to multiple environments. However, these processes differ across bacterial strains from the same species, and across different plant species or cultivars. In a competitive environment, additional risk factors are the plant microbiome phyllosphere and the plant responses; both factors directly modulate the survival of the pathogens on the leaf’s surface. Understanding the mechanisms involved in bacterial attachment to, colonization of, and proliferation, on fresh produce and the role of the plant in resisting bacterial contamination is therefore crucial to reducing future outbreaks.

## 1 Introduction

Over the last couple of decades, an increase in the consumption of fruits and vegetables has been recommended by multiple governments and the World Health Organisation (WHO) (Rome Declaration on Nutrition and Framework for Action, 2014; Recommendation 21). Fruit and vegetables provide an accessible source of nutrients and fibre to consumers and are associated with a range of health benefits. Indeed, a daily intake of fruit and vegetables is recommended to reduce chronic illnesses, including heart disease, cancer and diabetes (World Health Organization, 1990). Between 1960 and 2019, fruit and vegetable consumption worldwide increased from 60 to 140 kg per capita per year (Food and Agriculture Organization of the United Nations, 2019). In parallel with this increase in consumption, there is evidence of increasing foodborne illness outbreaks, particularly across North America; the percentage of outbreaks attributed to fruit and vegetable consumption doubled from 8% between 1998 and 2001 to 16% between 2010 and 2013 (Bennett et al., 2018). A systematic review indicates substantial increases in foodborne illness outbreaks relating to bacteria in the USA between 1999 and 2019, although for the EU, the tendency is not as clear (Aiyedun et al., 2021). Whilst the consumption of fruit and vegetables remains relatively safe, the agri-food industry faces multiple risks of introducing foodborne pathogens to fresh produce at all stages within its life cycle (Carstens et al., 2019). This risk is highest in minimally processed fresh produce that does not include a ‘kill’ step to reduce microbiological load and is usually consumed raw.

Foodborne illness outbreaks can be caused by a range of microbiological agents, including bacteria, parasites, viruses, fungi and mycotoxins. In 2010, the WHO attributed norovirus to 120 million of a total of 600 million global cases of illnesses caused by foodborne pathogens (Havelaar et al., 2015). Norovirus was the primary contaminant responsible for foodborne illness outbreaks related to fruit and vegetables in both the USA (59%) and the EU (53%) between 2004 and 2012 (Callejón et al., 2015). However, bacterial pathogens are the second major contributor to outbreaks, representing 36% and 42% of the outbreaks associated with fruit and vegetables in the USA and EU, respectively, between 2004 and 2012 (Callejón et al., 2015). Three bacterial species commonly associated (focus of this review) are *Salmonella enterica, Escherichia coli* and *Listeria monocytogenes*, which were responsible for 82% of all hospitalisations and deaths caused by foodborne illness outbreaks in the USA between 2009 and 2015 (Dewey-Mattia et al., 2018), although other bacteria including *Bacillus cereus, Vibrio cholerae, Campylobacter* spp., *Shigella* spp. and *Clostridium* spp. have also been reported.

### 1.1 Salmonella

*Salmonella* was the leading cause of bacterial foodborne illness both in Europe, between 2007 and 2011, and in the USA, between 2006 and 2015 (Ölmez, 2016), as well as the leading cause of hospitalisation and deaths in the USA by known foodborne pathogens (Scallan et al., 2011). Moreover, in the EU, food surveillance sampling reported that up to 0.84% of ready-to-eat (RTE) fruits and vegetables were positive for *Salmonella* (EFSA, 2018a). *Salmonella* can be subdivided into two species: *S. bongori*, which is rarely associated with human disease, and *S. enterica* (Hohmann, 2001; Leekitcharoenphon et al., 2012), which is the pathogenic species. *S. enterica* is subdivided into more than 2500 serovars, that differ in their surface characteristics (Lipopolysaccharide O antigen and flagella: H antigen). The distribution of these serovars on fresh produce seems to depend on geography: *S. enterica serovar* Newport was the serovar associated with most fruit and vegetable-related outbreaks in the USA, whilst in the EU, *S. enterica* serovar Enteritidis was the most common serovar associated with salad (Bennett et al., 2018; Callejón et al., 2015). Interestingly, the serovars identified from fruits and vegetables differ greatly from the serovars found associated with farm animals (Ferrari et al., 2019), suggesting certain serovars may be better adapted to colonise plants than others. Indeed, serovars even differ in their ability to adhere to and colonise different plant species. This has been shown experimentally, with *S*. Enteritidis, Typhimurium and Senftenberg adhering more to basil than *S*. Arizona, Heidelberg or Agona (Berger et al., 2009a), and *S*. Tennessee adhering more to lettuce than *S*. Negev (Patel and Sharma, 2010). The overall level of adhesion can also vary depending on the species of vegetable. For example, cabbages have been reported to support less *Salmonella* adhesion than lettuce (Patel and Sharma, 2010). These differences can also be observed at the plant cultivar level, with *S*. Typhimurium showing increased adhesion to cultivar “Nelly” compared to “Cancan” lettuce (Klerks et al., 2007) and greater adhesion to “Romaine” compared to “Iceberg” lettuce (Patel and Sharma, 2010). Altogether, this suggests serovar-specific attachment mechanisms cause specific serovars to be more likely to contaminate certain fresh produce. This is supported by data from outbreak reports between 2006 and 2023 in the USA, which show that a range of *Salmonella* serovars can contribute to outbreaks (Table 1). Sprouted vegetables were a common vector for *Salmonella* spp., as well as papaya (Hassan et al., 2019), melon/cantaloupe (Chan et al., 2023), cucumbers and tomatoes (Gurtler et al., 2018). Cucumber contamination included a large-scale outbreak of *S*. Poona in the USA, which led to 907 cases across 40 states and six fatalities (Laughlin et al., 2019). *Salmonella* also poses a significant public health risk in the EU, including an outbreak of *S*. Strathcona in 2011 which was traced back to ‘datterino’ tomatoes, responsible for 43 cases in Denmark, and 28 cases across Germany, Italy, Austria, and Belgium (Müller et al., 2016). Cucumbers were again implicated as a vector in Europe: between 2016 and 2017, 147 cases of *S*. Agona were reported across five EU countries linked to the consumption of products containing cucumbers, although there was insufficient microbiological evidence to definitively confirm this (EFSA, 2018b).

### 1.2 Escherichia coli

*E. coli* is a species of almost exclusively non-pathogenic bacteria that is part of the commensal flora of mammals, and contributes to the digestion of food and the production of vitamin K (Review in Martinson and Walk, 2020). However, certain strains can cause diarrhoea, urinary tract infections, sepsis, and meningitis in humans (Leimbach et al., 2013). Diarrheagenic *E. coli* are broadly categorised into seven classes called pathotypes: enterotoxigenic (ETEC), enteropathogenic (EPEC), Shiga toxin-producing (STEC), enteroaggregative (EAEC), enteroinvasive (EIEC), adherent-invasive (AIEC) and diffusely adherent *E. coli* (DAEC) (Rojas-Lopez et al., 2018). These pathotypes are characterised by different somatic (O), flagellar (H) and capsular (K) surface antigens, and by the presence of specific virulence factors. Interestingly, the prevalence of foodborne illness outbreaks associated with *E. coli* on fresh produce is higher in the USA relative to the EU, accounting for 12.2% and 3.8% of outbreaks of bacterial foodborne illness, respectively (Callejón et al., 2015).

In the USA, STEC was the pathotype most associated with outbreaks of foodborne illness, predominantly belonging to serogroup O157:H7, which accounted for 92% of cases between 1998 and 2013 (Bennett et al., 2018). In 2015, a new highly pathogenic strain of O157:H7 emerged in England and Wales, which has been identified in patients and was associated with the consumption of prepacked salad leaves (Byrne et al., 2018). It is, therefore, critical to analyse the behaviour of these new strains in the environment, as well as their capacity to cause disease. Lettuce is commonly associated with foodborne illness outbreaks caused by *E. coli*, including several outbreaks between 2006 and 2020 in the USA (Table 1). As with *Salmonella, E. coli* is also commonly associated with consumption of sprouted vegetables (Table 1). One example includes a large-scale outbreak of *E. coli* O104:H4 in Germany, which was associated with the consumption of raw sprouts (lentil, alfalfa, fenugreek and adzuki bean), leading to 3816 cases and 54 fatalities (Frank et al., 2011; Buchholz et al., 2011). One of the largest outbreaks of *E. coli* occurred in 1996 in Japan, where contamination of white radish sprouts with *E. coli* O157:H7 traced from a single farm led to 9441 cases, and 12 fatalities (Michino et al., 1999). This example highlights the impact that a single source of enteric pathogens can have on a wide range of consumers.

### 1.3 Listeria monocytogenes

**Fig. 1:**
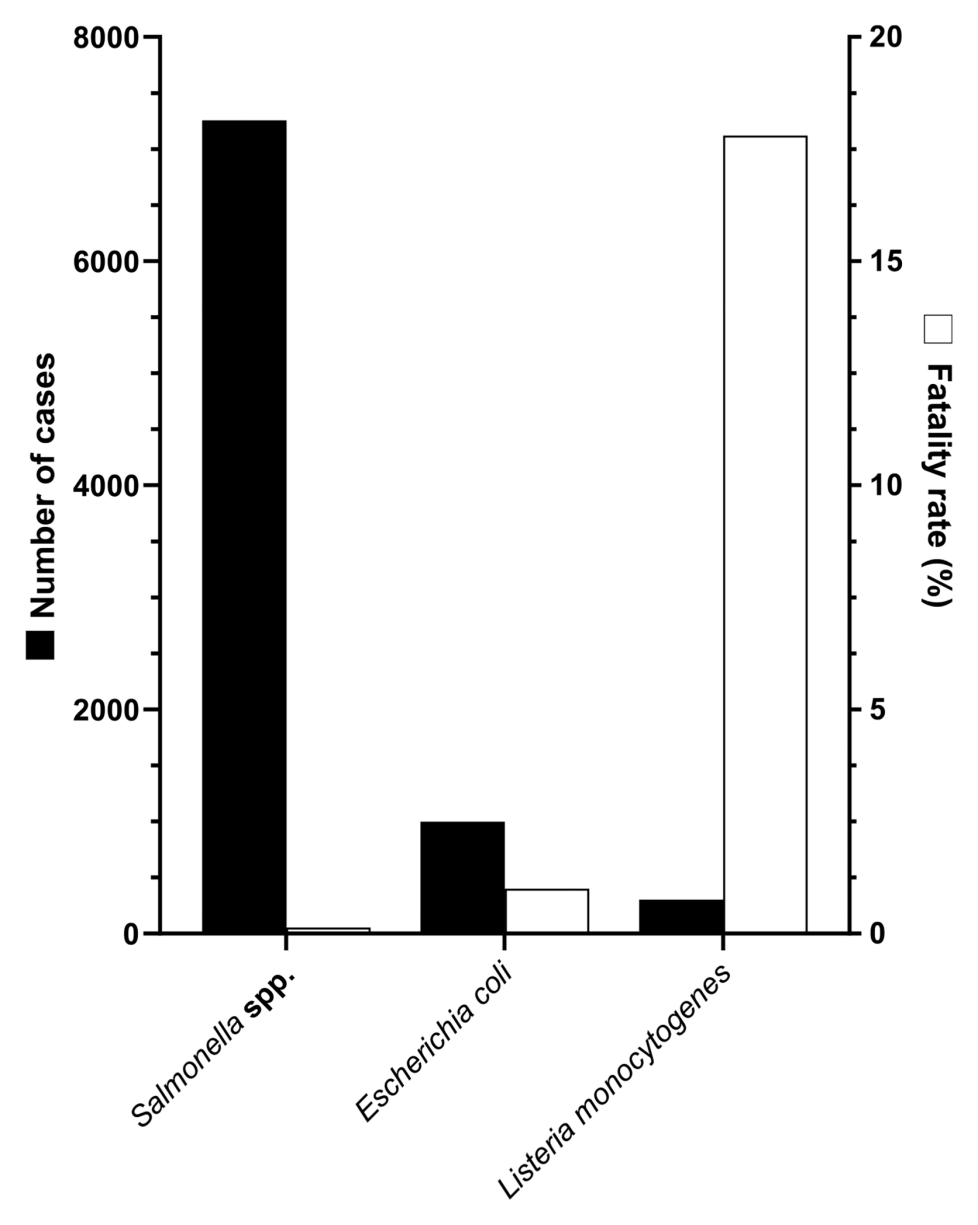
Number of cases and associated case fatality rates related to consumption of contaminated fruits and vegetables from CDC data, 2006–2023.

Whereas *Salmonella* and *E. coli* are the two leading causes of bacterial outbreaks linked to the consumption of fresh fruit and vegetables, *L. monocytogenes* has caused comparatively fewer outbreaks, but a greater cost for the food industry (Fig. 1). Listeriosis results in the highest case fatality rate of the three bacterial pathogens discussed here, and ranks as one of the most frequent causes of death due to foodborne illness (Behravesh, et al., 2011; Werber et al., 2013; The European Union One Health 2018 Zoonoses Report, 2019 Zoonoses Report, 2019). *L. monocytogenes* can be subdivided into at least 13 serotypes (similar to serovars), differing in their pathogenicity. Between 1998 and 2003 in the USA, serotype 1/2a caused eight outbreaks with a 45% hospitalisation rate and 7% case fatality rate, whereas 1/2b caused two outbreaks with a 60% hospitalisation rate but no fatalities (Cartwright et al., 2013). However, serotype 4b is responsible for the majority of human listeriosis outbreaks, and led to 10 outbreaks, with a hospitalisation rate of 70%, and a case fatality rate of 13%. Several national outbreaks of *L. monocytogenes* have been reported in the USA associated with contaminated fruit and vegetables (Table 1). For example, in 2011, a multi-state outbreak of *L. monocytogenes* on cantaloupe melons from a single farm in Colorado led to 147 cases across 28 states, causing 143 hospitalisations and 33 deaths (McCollum et al., 2013). Similarly, an outbreak in 2014 led to 35 cases across 12 states including seven deaths and was linked to an apple packing factory (Angelo et al., 2017). In Europe, between 2013 and 2014, 32 cases of listeriosis associated with ready-to-eat salads were reported in Switzerland, for which serotype 4b was responsible (Stephan et al., 2015). Another outbreak of *L. monocytogenes* serotype 4b across five EU member states led to 47 cases and nine deaths and was linked to the consumption of frozen sweetcorn and other frozen vegetables (EFSA, 2018c).

This review will focus on sources of contamination of fresh produce with three of the major human enteric pathogens: *Salmonella* spp, *E. coli* and *L. monocytogenes*. We consider their mechanisms of attachment, how the plant responds to and can affect colonisation with these bacteria, and future perspectives for reducing contamination and disease outbreaks.

## 2 Contamination routes

**Fig. 2:**
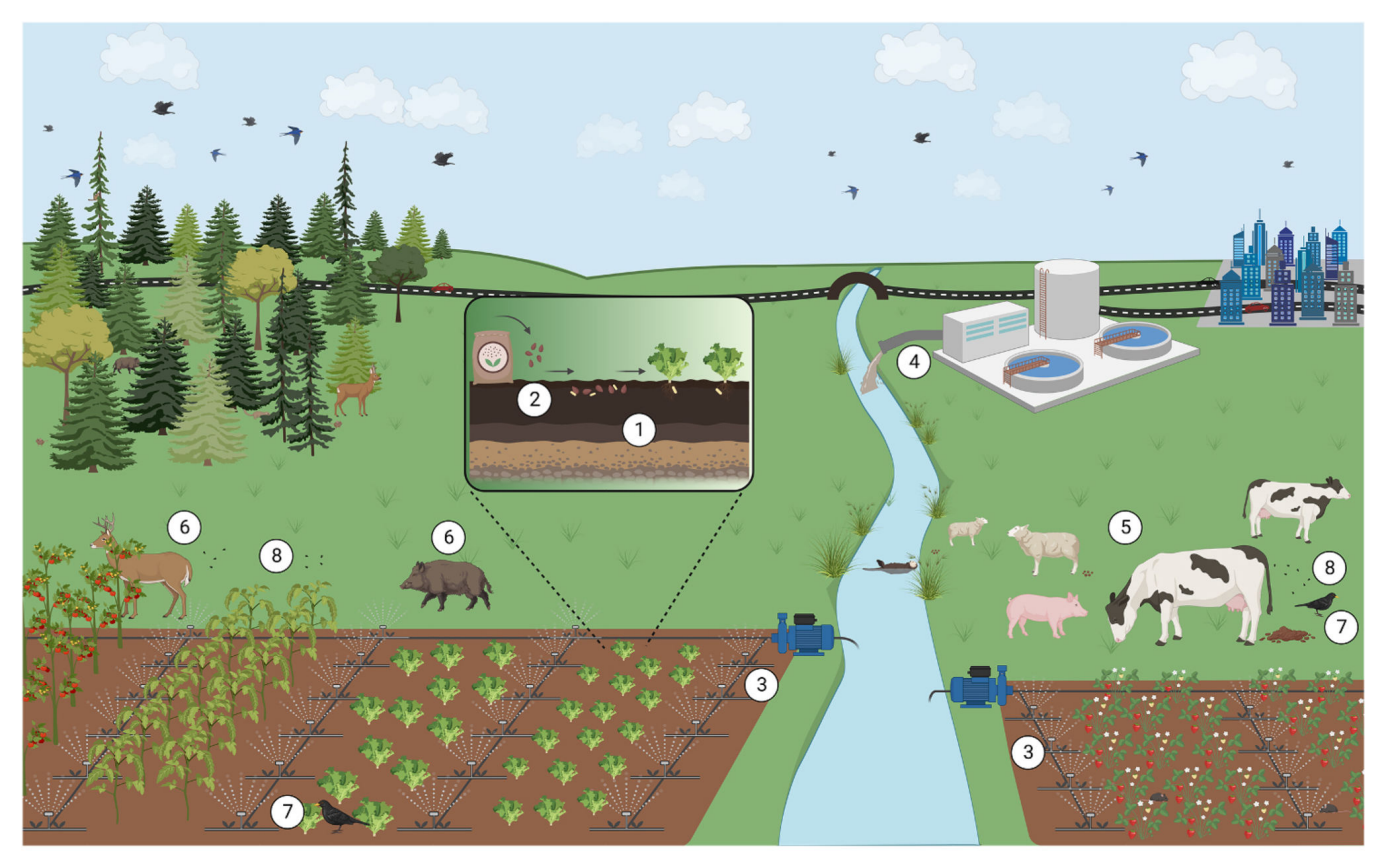
Overview of potential sources of contamination of fresh produce throughout the pre-harvest stages. Colonisation of fruits and salads with human enteric pathogens can derive from multiple origins including soil contamination (1), seeds (2), irrigation system (3) and grey/blackwater (4), domestic (5) or wild mammals (6), birds (7) and insects (8).

To reduce outbreaks of foodborne illness, an understanding of potential routes which introduce bacterial contaminants into the fresh produce supply is crucial. This is complicated by the fact that fruit and vegetables can be contaminated at multiple points in the supply chain (Fig. 2).

### 2.1 Pre-harvest contamination

During the crop cycle, multiple sources of contamination have been identified from soil, seeds, irrigation water and domestic and wild animal faecal matter. Each of these sources can enable bacteria to establish themselves on the growing crops, where they can survive and multiply under favourable conditions (Park et al., 2012).

One of the first sources of contamination is the soil, especially if sites used for propagating fresh produce were previously used for animal production, waste disposal, or if manure was applied as fertiliser (Uyttendaele et al., 2015). However, whereas most enteric pathogens are hosted by animals, *L. monocytogenes* is a ubiquitous environmental bacterium, which is frequently isolated from soil (reviewed in Vivant et al., 2013; Smith et al., 2018), even without obvious sources of animal-derived contamination. Below ground parts of the plant will come into direct and close contact with the soil, hence root vegetables (e.g. carrots) eaten raw and not carefully washed or peeled may be more vulnerable to contamination. Extreme weather events can also lead to foodborne illness outbreaks, including flooding (Castro-Ibáñez et al., 2015; Bergholz et al., 2016). With the increase in global warming, dust events are becoming an additional risk for contamination. In 2018 in Australia, a dust storm was suggested to have contributed to the contamination of rock melons with *L. monocytogenes* (NSW Department of Primary Industries, 2018). Similarly, in 2022 in the UK, a dust storm during a very dry period was associated with the contamination of salad crops with STEC carried from an animal farm nearby (FSA, personal communication).

Contamination of fresh produce grown in fields can arise both when leaves come into direct contact with soil, but also splash events can transfer pathogens in the soil onto the leaves, which has been shown experimentally for *Salmonella* (Cevallos-Cevallos et al., 2012; Lee et al., 2019) and *Listeria innocua* (Girardin et al., 2005). This risk of splash contact by enteric pathogens may be increased by their ability to persist for long periods in soil: *S*. Typhimurium can persist for up to 231 days (Islam et al., 2004b), *E. coli* O157:H7 for up to 217 days (Islam et al., 2004a), and *L. monocytogenes* for up to 360 days in soil microcosms (Piveteau et al., 2011). However, these survival times in soil can be influenced by soil characteristics, including physical soil characteristics, with *Salmonella* persistence greater in loamy rather than sandy soil (Jechalke et al., 2019). Moreover, the presence of other microbes in soil can also influence enteric pathogen persistence, with a reduction in survival of *Salmonella* when soil prokaryote diversity is lower (Schierstaedt et al., 2020). The ability of *L. monocytogenes* to colonise roots can also be impacted by other rhizobacteria present in the soil. For example, the presence of one bacterial species (*Pseudomonas simiae*) enhanced *L. monocytogenes* colonisation of plant roots, and ten (nine *Pseudomonas* spp. and one *Burkholderia* spp.) inhibited its association (Schoenborn et al., 2021). Similarly, plants can also influence *Salmonella* distribution in soil, with migration of *Salmonella* towards root exudates of both *Arabidopsis* and lettuce (Karmakar et al., 2019; Klerks et al., 2007), suggesting plant root exudates may act as attractants for human enteric pathogens.

While soil can be a source of contamination for enteric pathogens, the possibility of seed contamination cannot be excluded. Two main mechanisms have been identified: (i) the attraction of enteric pathogens in the soil towards germinating seeds and (ii) the sowing of pre-contaminated seeds. Once contaminated seeds germinate, pathogens can then spread to contaminate the edible material. This explains why sprouted seeds have been responsible for several *Salmonella* and *E. coli* outbreaks (e.g. Mahon et al., 1997; Frank et al., 2011; Buchholz et al., 2011; Michino et al., 1999), with bacterial proliferation potentially facilitated by the sprouting process, leading to pathogen enrichment (National Advisory Committee on Microbiological Criteria for Food, 1999). Indeed, in the laboratory, both *Salmonella* and *E. coli* can attach directly to seeds, although greater populations of *Salmonella* were generally supported (Cui et al., 2017; Liu et al., 2018). Inoculating a range of vegetable seeds (fenugreek, alfalfa, tomato, and lettuce) with *Salmonella* and *E. coli* resulted in recovery of the pathogens from roots, stems, and cotyledons of the vegetables (Liu et al., 2018), providing evidence that contaminated seeds can develop into contaminated plants. Similarly, *L. monocytogenes* inoculated onto seeds was recovered from 7-day old *Arabidopsis* seedlings (Milillo et al., 2008) and on 60-day old lettuce plants grown from inoculated seeds (Shenoy et al., 2017). Whilst these findings have been demonstrated under laboratory conditions, the level of seed contamination in agro-industrial settings is still unknown, as is the occurrence of such events in farms.

The risk of enteric pathogen contamination from seeds highlights the need for effective decontamination methods to reduce pathogen populations, without compromising seed germination. Chemical treatments have been recommended by the U.S. Food and Drug Administration (FDA), including treatment of seeds with 20,000 ppm hypochlorite. However, this treatment may not fully eliminate the presence of enteric pathogens, as previous outbreaks have occurred even after chemical treatment (Proctor et al., 2001), suggesting other treatments are therefore required. For example, the combination of heat, acetic acid, and H_2_O_2_ in mung bean seed treatments reduced populations of all three enteric pathogens by a factor of 1000 (Trząskowska et al., 2018). Similarly, non-thermal methods, including treatment with chlorine dioxide gas, ozone gas, or e-beam irradiation, all significantly reduced populations of *Salmonella* and *E. coli* on tomato, lettuce, and cantaloupe melon seeds, although cantaloupe seed germination was compromised following chlorine dioxide treatment (Trinetta et al., 2011). A comprehensive meta-analysis comparing chemical, biological and physical treatments to the FDA-recommended treatments indicates treatment of seeds with heat and high pressure (physical) can sanitise seeds potentially more effectively than through the recommended 20,000 ppm calcium hypochlorite (Ding et al., 2013). Following the outbreaks of *E. coli* O104:H4 in Europe in 2011, regulatory bodies updated policies for foodstuffs to include specific legislation regarding sprouts and seeds intended for the production of sprouts, ensuring they are produced in a hygienic manner. Voluntary labelling of bags of romaine lettuce, including date and location of harvest for improved traceability, has also been recommended by the FDA following a 2018 outbreak, enabling sources of outbreaks to be traced more easily (FDA Statement, 2018).

Another well-known source of contamination is irrigation water, applied directly to crops during agricultural production. Water from rivers and lakes can introduce enteric pathogens on crops through contamination via runoff of sewage, soil, or animal faecal matter. This is becoming a particular issue with increased poor river quality due to sewage released from urban areas or farm runoff water. In 2050, it is estimated that 68% of the world’s population will be urban compared to only 30% in 1950 (United Nations, 2018). This large increase, associated with poor management or lack of investment in ageing sewage treatment plants, is associated with an increase of waste released into the environment, and contamination with enteric pathogens that can survive in this water for prolonged periods of time. In addition to urbanisation, global warming is exacerbating the lack of good-quality water availability. With increasing global temperatures, water scarcity is becoming a growing issue, particularly in lower-income countries which must therefore rely on lower quality water sources for irrigation. As such, crops are often irrigated with reused grey/blackwater; water which has been affected by domestic, industrial or commercial use, with an estimated 10% of the global population consuming agricultural products cultivated with treated wastewater (Victor et al., 2008). However, if this wastewater is not properly treated, it could pose a contamination risk (Papadopoulos et al., 2022). Several studies have reported the presence of enteric pathogens on crops irrigated with contaminated wastewater (Castro-Rosas et al., 2012). Poor water quality is not only limited to countries with low income (Abraham, 2011); high-income countries are facing similar issues. Examples of outbreaks due to contaminated irrigation water include a 2005 outbreak of S. Newport in the USA which was traced back to a pond in Virginia, used to irrigate tomatoes (Greene et al., 2008), outbreak strains of *E. coli* O157: H7 caused by romaine lettuce consumption in the USA (Bottichio et al., 2020), and watercress consumption in the U.K. (Jenkins et al., 2015), which were isolated from irrigation water adjacent to domestic cattle farms. Subsequently, the Leafy Green Food Safety Task Force recommended increasing buffer zones between concentrated animal feeding operations and farms where leafy greens are grown (Bottichio et al., 2020). In addition to the level of contamination of the source, the system of irrigation water will also impact the level of contamination since different regimes of irrigation water application affect the contact of irrigation water with edible crop material. Laboratory experiments reproducing irrigation by overhead sprinklers, which apply irrigation water directly to foliar material, showed greater recovery of *E. coli* compared to drip and furrow irrigation systems in both lettuce (Fonseca et al., 2011; van der Linden et al., 2013) and spinach (Mitra et al., 2009). Interestingly, this effect does not seem to apply to *Salmonella* where no difference was observed (Van der Linden et al., 2013). Taking all these factors together, irrigation water poses a risk of contamination of fresh produce with enteric pathogens, which could be reduced by monitoring the presence of pathogens, or with mitigation strategies to reduce microbial load. These include removal of debris from irrigation water, filtration, chlorination, electrolysis, chemical oxidation, UV treatments and irradiation (Banach and Van Der Fels-Klerx, 2020). Simple measures like exclusion fences restricting livestock access to streams have also been shown to reduce *E. coli* populations in the water (Bragina et al., 2017). General principles have been proposed relating to the microbiological safety of wastewater and include execution of and response to sanitary surveys, maintenance of irrigation water reservoirs and distribution systems, adequate water treatments, disinfection of irrigation water, and faecal indicator tests to monitor water quality (Uyttendaele et al., 2015). Whereas all these solutions have been shown to decrease the level of water contamination efficiently, their costs remain too high to be applicable in low-income countries.

Another possible route of pre-harvest contamination, but probably the least manageable, is linked to animals. Animals are a common reservoir of enteric pathogens and can be either the source of contamination via their faeces which can be shed into soil, water or directly onto the foliage, or the vector of numerous pathogens, carrying pathogens from one area to another. The main reservoir for *E. coli* O157:H7 is in the intestine of healthy cattle (Wells et al., 1991), and both *Salmonella* (reviewed in Gopinath et al., 2012) and *L. monocytogenes* (Lyautey et al., 2007) have also been detected in livestock. As well as domestic animals, wild animals pose a risk for produce contamination and are more difficult to control. *S. enterica* has been isolated from deer mouse, stray dog and coyote faeces in the Salinas Valley region of California, which produces around 91% of salads in California, highlighting a potential risk for future outbreaks related to leafy salads (Kilonzo et al., 2013; Jay-Russell et al., 2014). Birds may also act as longer distance routes of transmission of pathogens and have been shown to be potential vectors for all three pathogens (Wallace et al., 1997; Pedersen and Clark, 2007; Fenlon, 1985; Navarro-Gonzalez et al., 2020; Smith et al., 2022). This risk of contamination of fresh produce by enteric pathogens from animal faeces is evidenced by outbreaks of *E. coli* O157:H7 following the consumption of strawberries contaminated with deer faeces in Oregon (Laidler et al., 2013), and spinach contaminated with feral swine faeces in Canada (Jay et al., 2007). As a result of such outbreaks, regulatory bodies have made several recommendations to mitigate risks, including the installation of wildlife fences and rodent traps surrounding fields, to reduce wildlife intrusion (Beretti and Stuart, 2008). A systematic review highlights several suggestions to mitigate food safety risks in agricultural regions, whilst maintaining biodiversity and improving farmer livelihoods (Olimpi et al., 2019).

Manure from domestic animals and slurry are often applied to agricultural soils as a form of fertiliser, which, when inadequately composted, can, in fact, provide a source of contamination and has led to previous outbreaks of *E. coli* in lettuce and spinach (CDC, 2018; Grant et al., 2008). Quantitative microbial risk assessment models highlighted the risk of human exposure to *L. monocytogenes*, and pathogenic *E. coli* when manure or slurry are released onto arable lands without proper treatment such as pasteurisation (Nag et al., 2022). This may pose a greater risk as shifts towards organic agricultural practices are favoured. Livestock are also often reared adjacent to arable land for fruit and vegetable production, meaning untreated manure or contaminated surface water could also come into contact with crops.

An additional risk factor that has been overlooked until recently is the plant-microbiome phyllosphere. Leaf phyllospheres harbour a diverse and dynamic community of microorganisms. These microorganisms play essential roles in plant health and development, nutrient cycling, and protection against plant pathogens (reviewed in Sohrabi et al., 2023) but could also either facilitate or prevent the colonisation of the leaves or fruit by *Salmonella, E. coli* or *L. monocytogenes*. This inhibition may be related to space exclusion, nutrient competition or active elimination (e.g. acid production, antimicrobial peptides). Where the bacterial microbiota within the plant could inhibit the growth and persistence of *Salmonella, E. coli* or *L. monocytogenes* (Lopez-Velasco et al., 2012; Carlin et al., 1996), different genera or species have been shown to have different effects. Although *Flavobacterium* increased spinach colonisation with *E. coli* (Lopez-Velasco et al., 2012), *Enterobacter cloacae* reduced *E. coli* and *L. monocytogenes* colonisation on lettuce (Jablasone et al., 2005). Interestingly, lettuce grown under glasshouse conditions had distinct phyllosphere microbiota compared to field-grown lettuce. However, microbial community transfer from the field-grown to lab-grown lettuce did not change *E. coli* survival (Williams and Marco, 2014). In addition, plant pathogens could also impact colonisation by human pathogens. For example, *Xanthomonas hortorum* pv. gardneri infection of tomato leaves has been shown to increase survival of S. *enterica* (Dixon et al., 2022).

Whilst knowledge about the influence of phyllosphere microbiota diversity on human pathogen colonisation is increasing, the field suffers from difficulties related to the culture of the microbiota and field experimentation.

### 2.2 Post-harvest contamination

Post-harvest operations, including storage, preparation and packaging, can cause enteric pathogen contamination if not controlled correctly in accordance with good manufacturing practices (Ölmez, 2016). Hazard Analysis and Critical Control Point (HACCP) principles can be put in place to identify points which are at risk of introducing hazards along the production line. For example, monitoring of water quality used for salad washing, daily sanitation of machinery, as cutting is a critical point, and critically temperature control (Calonico et al., 2019). For this, it is important that companies develop flow charts and decision trees detailing their processes, which can be subsequently assessed.

At the raw material stage, leaf damage can frequently occur following harvesting, which can alter the phyllosphere environment of the leaves and provide sites of adhesion for pathogens. For example, lesion areas on leaves increased during the processing of leafy greens from field to bag, leading to an increase in the relative abundance of bacteria belonging to *Pseudomonadaceae* and *Enterobacteriaceae* on spinach and chard leaves (Mulaosmanovic et al., 2021). Mechanical damage of lettuce leaves resulted in the support of higher numbers of *E. coli* (Brandl, 2008; Aruscavage et al., 2008) and *Salmonella* (Van der Linden et al., 2013), and was also associated with higher levels of invasion of GFP-tagged *E. coli* into rocket and chard tissue (Hartmann et al., 2017), and spinach tissue (Mulaosmanovic et al., 2021). The cooling process used to remove field heat may also contribute to contamination levels. Vacuum cooling is a common practice and involves prior spraying with water to reduce weight loss (Pyatkovskyy et al., 2021). Although this rapid cooling can improve salad quality, which may, in turn, reduce bacterial pathogen growth, it could also result in increased pathogen contamination through the formation of aerosols. As well as during the harvesting stage, the chopping stage of fresh produce could also introduce pathogens. Cut edges of lettuce have supported higher levels of *L. monocytogenes* (Gorski et al., 2021). Mechanical damage to plants also includes the packaging of produce. The release of water and nutrient-rich exudates caused by rupturing the protective barrier of the leaves (epidermis) can lead to the accumulation of juices in salad bags, which supported proliferation of *Salmonella* (Koukkidis et al., 2017). Minimising pre- and post-harvest damage is therefore critical to reduce contamination by enteric pathogens. Removing damaged leaves, and consuming bagged salad on the day of purchase, can help to alleviate these risks.

Following raw material harvesting and storage, the preparation stage of processing occurs, which involves several steps. Washing leafy produce after harvest is crucial for removing soil debris but may also become a source of contamination and therefore contribute to post-harvest contamination of fresh produce. For example, wash water was the source of contamination of melons with *Salmonella* in the Rio Grande River Valley outbreak (Gagliardi et al., 2003). Experimental evidence highlights this risk of transmission from a single source during this step: washing a batch of lettuce in which only 5% of heads were contaminated with *Salmonella*, resulted in a homogenous distribution of the pathogen across the entire batch (Pérez-Rodríguez et al., 2014). Washing typically consists of three stages in three separate tanks; the first to remove soil debris, the second to prevent cross-contamination through disinfectant treatment, and the third using non-chlorinated rinse water to remove the disinfectant (Gil et al., 2015). Water alone has been shown to be ineffective in reducing *E. coli* levels relative to unwashed controls (Holvoet et al., 2014), which emphasises the importance of using alternative post-harvest decontamination methods. For example, UV-A light and benzoic acid can also reduce the bacterial population of *E. coli* on spinach, without causing colour defects on the foliage (Ding et al., 2018), and UV-C stress can also be used to reduce *L. monocytogenes* contamination on lettuce leaves (Kyere et al., 2021). Other alternatives to the use of chlorinated compounds include irradiation, pulsed light, ozone, advanced oxidative processes and gas plasma, with varying degrees of log count reduction reported for the key bacterial pathogen contaminants (reviewed in Murray et al., 2017). These alternatives need further evaluation for future use by the industry to replace the use of chlorinated compounds.

The preparation of fresh fruit salads also entails potential risks of contamination. Specifically, bacterial loads resident on the peel or rind of fruits can be accidentally transferred to the flesh when chopping (Willis et al., 2016; Luciano et al., 2022) as was found in a recent contamination of watermelon imported into the UK (Chan et al., 2023). Melons and watermelons are of particular concern due to the relatively low acidity of the flesh that favours microbial growth (Luciano et al., 2022). In addition, mechanical damage caused during the processing steps such as washing, sanitising, peeling and chopping can result in softening which favours microbial growth (Zhao et al., 2022).

In addition to leaf damage, enteric pathogens can also be introduced from contaminated surfaces during the processing. Between 2013 and 2014, an outbreak of *L. monocytogenes* in Switzerland was traced back to a specific product-feeding belt which fed the product into a colour sorter. The belt may not have been effectively sanitised due to design flaws, meaning the belt was not fully accessible for daily disinfection procedures (Stephan et al., 2015). The ability of the pathogens to attach and colonise surfaces is also directly related to the formation of a biofilm by the bacteria. Following initial adhesion, bacteria can form a matrix created by the secretion of extracellular polymeric substances (nucleic acids, exopolysaccharides, and proteins). This matrix physically links bacteria together within a colony and enables adhesion to surfaces protecting the bacteria from harsh environmental conditions (Costerton et al., 1999; Yaron and Römling, 2014). Biofilm formation is directly related to the bacterial strain, nutrient availability and temperature. Previous studies have shown that different *S*. Typhimurium and *L. monocytogenes* strains form biofilms on a range of surfaces including on polystyrene, polycarbonate, stainless-steel, glass and rubber (Mafu et al., 1990; Patel et al., 2013). Interestingly, 30-day old biofilms of several strains of STEC on stainless steel could transfer onto fresh lettuce at 25 °C but not 10 °C (Adator et al., 2018). This shows that adhesion and transfer of STEC biofilms from surfaces to fresh produce can occur, but the risk is reduced under refrigerated temperatures. These studies also highlight the need for effective sanitation of surfaces during the processing stage.

Finally, several studies indicate that the storage of produce can influence pathogen proliferation and survival. Enteric pathogens have been shown to survive on the surface of fruits, including apples (Alegre et al., 2010b; Buchanan et al., 1999; Burnett et al., 2000; Cuzzi et al., 2021; Kenney et al., 2001; Liao and Sapers, 2000; Sheng et al., 2017), avocados (Cabrera-Díaz et al., 2022), strawberries (Flessa et al., 2005), peaches and nectarines (Alegre et al., 2010a; Kuttappan et al., 2021), cantaloupe melon (Nyarko et al., 2016), mango and papaya (Luciano et al., 2022). Survival of pathogens on fruit depends on a range of factors, including intrinsic and environmental factors. An important intrinsic factor is the pH of the flesh. The flesh of most fruit is acidic which tends to inhibit microbial survival and growth, however, melon and watermelon flesh typically has a pH of around 6 and has been found to support higher populations of *L. monocytogenes* than other fresh-cut fruits including pears (Colás-Medá et al., 2017), mango, and papaya (Luciano et al., 2022). Of the environmental factors that can be controlled in the supply chain, temperature has been the most commonly studied factor in reducing the growth of enteric pathogens, and an efficient and highly controlled cold chain is currently central to reducing fresh-cut produce spoilage. In the case of *E. coli* and *S*. Typhimurium, no proliferation was observed when pathogens were inoculated at 5 °C, on both apples and peaches (Alegre et al., 2010a; Alegre et al., 2010b). Similarly, *S*. Typhimurium demonstrated no significant growth at 4 °C on fresh-cut dragon fruit (Sim et al., 2013). *L. monocytogenes* also demonstrated reduced proliferation on apples when stored at 10 °C or below (Sheng et al., 2017), and strawberries when stored at 4 °C (Flessa et al., 2005), whereas on mango, melon, papaya and a fruit mix, no increase in *L. monocytogenes* populations was observed (Luciano et al., 2022). A meta-analysis of *L. monocytogenes* growth and survival on intact produce demonstrated that both the storage temperature and the commodity influenced pathogen growth and survival, with produce stored at ≥20 °C showing the highest growth rates (Marik et al., 2020). The temperature-dependent survival of pathogens on produce may also differ amongst pathogens. For example, *L. monocytogenes* populations significantly increased on whole and sliced cucumbers stored at ~4 °C, although *Salmonella* populations significantly decreased at the same temperature (Bardsley et al., 2019a). Taken together, these data demonstrate the importance of temperature control to reduce contamination of produce by enteric pathogens. Whilst enteric pathogens may not proliferate at colder compared to more ambient temperatures, they can still survive on produce and therefore may pose a risk.

## 3 The interaction of bacteria with the plant surface involves attachment, survival/colonisation and internalisation

**Fig. 3:**
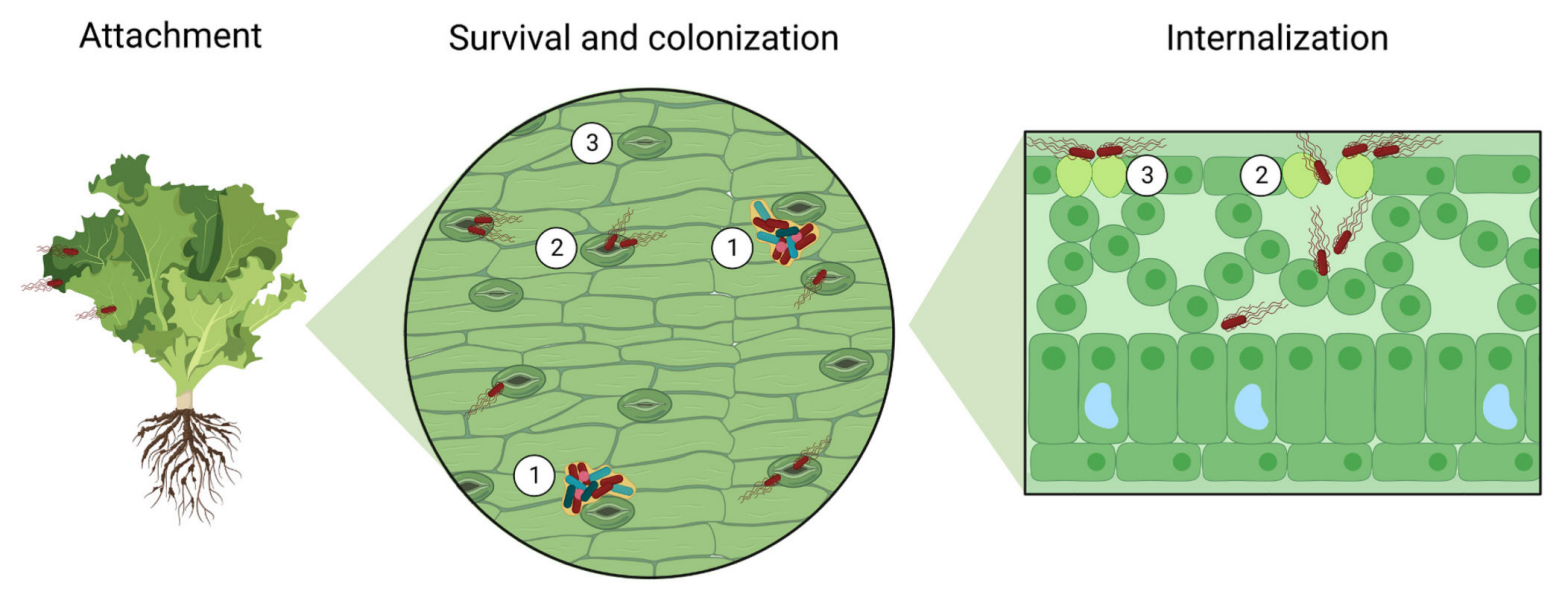
Overview of the stages of contamination of enteric pathogens of leaves, via attachment, colonisation/survival and internalisation. Following initial attachment to the leaves, pathogens will colonise the surface by producing different molecules including biofilms (1). Whereas some bacteria can attach to the stomatal cell (2) and invade the internal cavity, some trigger plant immune responses inducing stomatal closure (3), Reactive Oxygen Species (ROS) and ethylene production decrease the survival of the pathogens.

Outbreaks of foodborne illness associated with fruits and vegetables raise questions about the interaction between microbes and plants, as enteric pathogens are not usually considered part of the phyllosphere of leaves (Lim et al., 2014) or on fruit surfaces. Plant surfaces are stressful environments for enteric pathogens, since they are nutrient-poor compared to the gut of their usual warm-blooded hosts. Moreover, the micro-organisms are facing fluctuations in temperature, solar radiation, wind and rainfall, as well as the presence of indigenous populations of bacteria in the phyllosphere, which may be better adapted to survival on the leaf or fruit surface. Here, we will focus primarily on the leaf surface as a more complete picture of interactions is available, although some of the principles may apply also to the surface of other plant organs of relevance to minimally processed produce such as seed and fruit surfaces. A general model of leaf colonization by bacteria considers three stages: 1) bacteria arrive on leaves and adhere to the leaf surface, 2) bacteria multiply and form aggregates, and 3) bacteria internalise through open pores (Yaron and Römling, 2014) (Fig. 3).

### 3.1 Attachment to plant surfaces requires several bacterial cell surface components

**Fig. 4:**
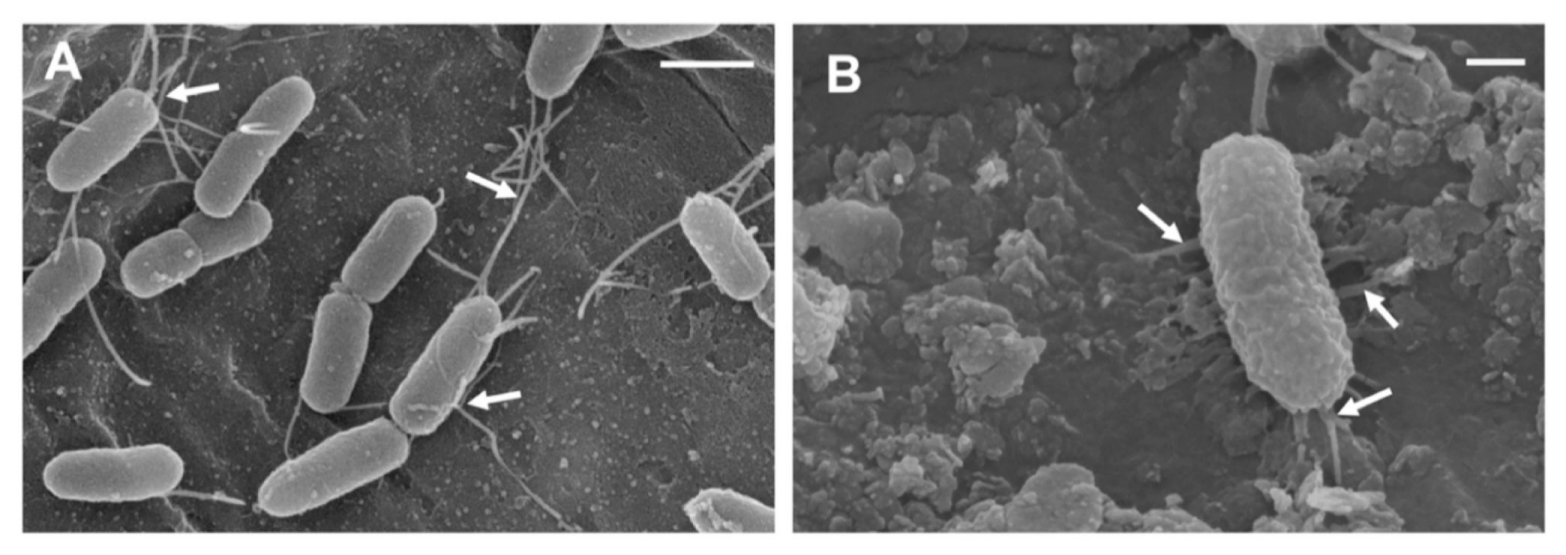
Bacterial attachment to salad leaves. Scanning electron micrograph showing adhesion of (**A**) *Salmonella enterica* serovar Typhimurium to spinach (Bar = 1 μm) and (**B**) *Listeria monocytogenes* to rocket (Bar = 200 nm). Arrows indicate adhesins potentially involved in the attachment to the leaf surface.

Bacterial attachment to fresh produce is the first stage of contamination on fruit and vegetables, preceding their colonisation and internalisation into edible plant tissue. Adhesion to fresh produce is probably an active process, since only viable cells of *S*. Typhimurium adhered to potato flesh (Saggers et al., 2008) and lettuce (Y. Kroupitski et al., 2009). In the case of *L. monocytogenes*, attachment can occur rapidly, within 1 second of contact with lettuce leaves (Kyere et al., 2019). The attachment of enteric pathogens to leaves is accomplished by several components of bacterial cell surfaces, including flagella, pili and fimbriae (Fig. 4A and B).

#### a) Flagella

Whilst flagella are primarily important for movement of bacteria, several studies indicate their potential role in adhesion to fresh produce. However, this adhesion is dependent on the pathogen serotype/serovar and plant species under investigation (Table 2). Indeed, deletion of *fliC*, the main subunit of the flagellum, reduced the adhesion of most pathogenic *E. coli* clones to leaves, including STEC on spinach (Saldaña et al., 2011; Xicohtencatl-Cortes et al., 2009; Nagy et al., 2016) and lettuce (Xicohtencatl-Cortes et al., 2009), and ETEC on rocket (Shaw et al., 2011a), although it did not appear to play a role in adhesion of STEC (Shaw et al., 2008) or EAEC (Berger et al., 2009b) to rocket. This difference may be explained by the presence of other adhesion mechanisms for these bacteria, including characteristic Aaf pili, which, upon deletion, showed reduced adhesion to rocket. The role of flagella in adhesion of *Salmonella* to basil leaves was also serovar specific: *S*. Senftenberg required flagella for adhesion to basil, but *S*. Typhimurium did not (Berger et al., 2009a). However, *Salmonella* express two types of flagella: phase 1 (*fliC*) and phase 2 (*fljB*) which are expressed interchangeably. It is, therefore, possible that *fljB* could play a role in *S*. Typhimurium adhesion. Deletion of both genes has been shown to reduce the adhesion of *S*. Typhimurium to *Valerianella locusta* leaves (corn salad) (Elpers et al., 2020), although deletion of *fliC*/*fljB* did not impede adhesion to tomato fruit (Shaw et al., 2011b) or leaves (Zarkani et al., 2020). As the leaves tested in this other study were different species, it is not possible to exclude that different mechanisms are adopted by *S*. Typhimurium depending on the plant species under investigation. Interestingly, different studies have shown a difference in adhesion between strains from the same pathotype of *E. coli* or serovar of *Salmonella* that have the same flagella type (*fliC*) (Shaw et al., 2011a,b; Berger et al., 2009a). However, to our knowledge, no study has investigated possible flagella mutations between these strains that could confer an increase in adhesion.

Unlike *E. coli* and *Salmonella*, the involvement of the flagella in fresh produce attachment of *L. monocytogenes* has received relatively little attention, although one study demonstrated a role of the flagellum (*flaA*) but not the flagellar motor (*motAB*) in adhesion of certain strains to alfalfa sprouts, broccoli, and radish. This suggests that the presence of flagella, but not their motility, are required for adhesion (Gorski et al., 2009). Interestingly, flagella are used by *L. monocytogenes* for attachment to radish plants, although only at temperatures below 30 °C (Gorski et al., 2003). This temperature-dependent role for flagella in adhesion has also been observed during adhesion to stainless steel, which occurred at 22 but not 37 °C (Vatanyoopaisarn et al., 2000). A fundamental outstanding research question is, therefore, whether the flagellum plays a role in adhesion of *L. monocytogenes* to salad leaves.

#### b) Fimbriae

Whilst the primary function of flagella is for bacterial movement, the primary role of fimbriae is considered to be adhesion. Fimbriae are hair-like appendages on bacterial cell surfaces. *Salmonella* and *E. coli* express different types of fimbriae; Tafi (Thin Aggregative Fimbriae) are expressed by *Salmonella*, and curli fimbriae are expressed by *E. coli*. Primarily, their role in adhesion is studied in the context of pathogenicity in human and animal health (reviewed in Rehman et al., 2019), although evidence also indicates a role in adhesion to plants. Expression of Tafi and curli fimbriae is controlled by aggregative fimbriae (*agf)* operons (*agfA to agfG*) (Collinson et al., 1996), and *csg* operons (*csgA* to *csgG*) (Hammar et al., 1995; Barnhart and Chapman, 2006) respectively. Interestingly, a role has been shown for *agfB* (encoding a subunit anchoring Tafi fimbriae to cell surfaces) in the adhesion of *S*. enteritidis to alfalfa sprouts, although *agfA* (encoding a major secreted subunit of Tafi fimbriae) was not involved in adhesion (Barak et al., 2005). Similarly, the equivalent gene of *agfA* in STEC (*csgA*, involved in curli expression) was not involved in adhesion to alfalfa sprouts (Torres et al., 2005) but was involved in adhesion to lettuce (Fink et al., 2012) and spinach leaves (MacArisin et al., 2012; Saldaña et al., 2011; Carter et al., 2016), indicating curli fimbriae may be involved in adhesion in a plant organ or plant-species-specific manner. Curli fimbriae are also involved in attachment of *E. coli* to stainless steel and glass surfaces (Carter et al., 2016).

As well as curli fimbriae, other fimbriae may be produced by different pathotypes of *E. coli*. A unique characteristic of EAEC is the presence of Aggregative Adherence Fimbriae (AAF), which appear to play a role in plant adhesion depending on the background strain and plant species. Deletion of two genes involved in AAF formation (*aafA* and *aggR*) impeded the ability of EAEC O44:H18 to bind the rocket leaf epidermis, which may explain why flagella did not appear to play a role in adhesion (Berger et al., 2009b). Contrastingly, deletion of *aggA* (encoding a major subunit of AAF) in the EAEC/STEC O104:H4 strain isolated during a major outbreak in 2011 did not impact adhesion to spinach (Nagy et al., 2016). This highlights the concept that extremely virulent strains may adopt several mechanisms in their adherence to fresh produce.

#### c) Type 3 secretion system

Type 3 secretion systems (T3SS) are a molecular syringe present on certain bacterial cell surfaces, whose primary role is the injection of effector proteins from the cytoplasm of bacteria into the plant cell, through the plasma membrane, which is surrounded by the plant cell wall (Büttner and He., 2009). As well as for effector delivery, T3SS also appear to have a crucial role in the adhesion of certain strains of *E. coli* to fresh produce. Whereas T3SS is conserved across many Gram-negative bacteria, the EPEC/STEC T3SS is unique due to the presence of a long filamentous extension (EspA filament) on top of the needle which mediates attachment to host cells. Deletion of the T3SS reduced adhesion of STEC to spinach (Xicohtencatl-Cortes et al., 2009) and lettuce (Saldaña et al., 2011), and seemed to eliminate adhesion of STEC to rocket (Shaw et al., 2008). Whilst deletion of a protein located at the tip of the T3SS *(*EspB) did not cause overall reduction in leaf attachment, there was a loss of stomatal aggregation of bacteria relative to wild-type, indicating a specific role for EspB in stomatal tropism (Shaw et al., 2008). A role for the T1SS and T3SS was also shown in the adhesion of *S*. Typhimurium to *Valerianella locusta* (Elpers et al., 2020).

#### d) Lipopolysaccharides

LPS are bacterial glycolipids found on the outer-membrane of gramnegative bacteria. LPS consist of three domains; lipid A, core oligosaccharide, and the O antigen (O–Ag). O-antigens are heterogenous in length, and depending on the number of repeated sugar units (between 16 and 100 units), they can occur in short (<16 units), long (16–25), and very long (>100) forms (Hölzer et al., 2009). The role of O-antigens in adhesion of STEC to plant surfaces is largely dependent on the plant species under investigation. They show a role in adhesion to lettuce leaves (Boyer et al., 2011), but not spinach leaves (Nagy et al., 2015) or alfalfa sprouts (Matthysse et al., 2008; Torres et al., 2005), although since different genes were investigated in each study it is difficult to draw broad conclusions (Table 2). Similarly, the presence of only very long O–Ag or only small O–Ag in *S. enterica* impairs binding to corn salad leaves (Elpers et al., 2020). Due to the high degree of structural heterogeneity of O antigen in *Salmonella* and *E. coli* (reviewed in Lerouge and Vanderleyden, 2002), it is difficult to cross-compare the role of O-antigen in adhesion to fresh produce across studies. O-antigen capsules, named due to their high degree of similarity to LPS_O-Ag_, may also play a role in adhesion of *Salmonella*. Indeed, deletion of a gene (*yihO*) encoding a transporter protein required for capsule assembly and transport led to a reduction in adhesion to alfalfa sprouts (Barak et al., 2007).

#### e) Other biofilm regulatory genes.

As well as a role in survival and colonization, biofilm regulatory genes appear to have a critical role in adhesion across *E. coli, Salmonella* and *L. monocytogenes* (Table 2). The most well-studied biofilm formation gene in the context of plant adhesion is the *ycfR* gene, encoding an outer membrane protein involved in stress regulation and biofilm formation. This gene has been shown to either promote adhesion of *S*. Typhimurium LT2 and *S*. Saintpaul to spinach leaves and grape tomato (Salazar et al., 2013a), or inhibit adhesion of *S*. Typhimurium ATCC14028 to cabbage (Kim and Yoon, 2019), highlighting the differences in adhesion mechanisms even within closely related isolates of *S*. Typhimurium. *ycfR* is also required for adhesion of STEC to lettuce (Fink et al., 2012). Other biofilm regulatory genes, including the *sab* autotransporter (Abe et al., 2020) and an enzyme (*pgaC*) involved in the production of the biofilm exopolysaccharide poly-β-1,6-*n*-acetyl-D-glucosamine (Matthysse et al., 2008), have been implicated in the adhesion of *E. coli* to rocket and alfalfa sprouts, respectively. Similarly, *Salmonella* genes involved in the biofilm formation (*sirA, yigA, bapA, siiE*) promoted adhesion to both spinach and grape tomato fruit (Salazar et al., 2013a), corn salad, and lettuce (Elpers and Hensel, 2020). Biofilm formation also seems to contribute to plant attachment for *L. monocytogenes*. Deletion of a Crp/Fnr family transcription factor *lmo0753*, which shows homology to a global factor required for biofilm formation, reduced levels of attachment of *L. monocytogenes* to both romaine lettuce and cantaloupe rind (Salazar, Wu, et al., 2013). Whilst biofilm formation is often considered as a mechanism for survival on surfaces, these studies indicate that several biofilm components appear also to play a role in initial adhesion across the three bacterial species. However, as biofilm formation by bacteria relies on multiple, complex regulatory processes controlled by several genes (reviewed in Yaron and Römling (2014)), cross-comparison of the mechanisms across different bacterial species should be performed with caution. A complete analysis of all the genes involved, for example, through systematic mutation studies and tested across different plant species, is urgently needed to understand which genes are important and whether the same mechanisms operate across the three different enteric species.

#### f) Cellulose

Cellulose is secreted by bacterial cells as a constituent of the biofilm matrix and may also be important in initial adhesion to plant leaves. Cellulose is synthesised by the bacterial cellulose synthase (Bcs) complex, which in most bacteria comprises two major subunits, BcsA and BcsB, as well as an outer-membrane protein, BcsC. Several studies highlight the role of the Bcs complex in the adhesion of *Salmonella* to fresh produce. BcsA is the catalytic subunit which synthesises cellulose and is required for optimal adhesion of *S*. Enteritidis to alfalfa sprouts (Barak et al., 2007), as well as for transfer of *Salmonella* to parsley via artificially contaminated irrigation water (Lapidot and Yaron, 2009). Interestingly, the role of this enzyme in adhesion appears to be temperature dependent, since there was a significant reduction in adhesion of *bcsA* mutants to plant cell wall models at 37 °C, but not 28 °C (Tan et al., 2016); this may be of relevance to the mechanism of adhesion in the field in warmer climates. Moreover, *Salmonella* mutants lacking the Bcs outer membrane protein, BcsC, showed a reduction in adhesion to tomato fruit disks (Shaw et al., 2011b). Whilst these studies suggest an important role for the cellulose synthase complex in adhesion of *Salmonella* to fresh produce, its role may not be as important in adhesion of *E. coli* to plant surfaces. Deletion of *bcsA* did not impair adhesion of STEC to spinach (MacArisin et al., 2012; Saldaña et al., 2011; Macarisin et al., 2013), although *yhjN* (synonymous with *bcsB*) mutants of *E. coli* were significantly impeded in their adhesion to alfalfa sprouts (Matthysse et al., 2008). However, introduction of a cellulose synthase gene into non-pathogenic *E. coli* K12 enhanced its ability to adhere to alfalfa sprouts. These studies highlight a critical role for *bcsA* in adhesion of *Salmonella* across several plant species, but not *E. coli*, whereas *bcsB* plays a role in adhesion of *E. coli* to sprouts. Further studies are needed to understand whether the role of *bscB* in *E. coli* is specific to alfalfa as a species, specific to the plant tissue, or whether other experimental factors are important. Finally, in *L. monocytogenes*, cellulose binding seems to be important in attachment to several different plant matrices: a putative cellulose binding protein (Lcp) was shown to be upregulated during attachment to lettuce, and deletion of the gene demonstrated a reduction in attachment not only to lettuce but also baby spinach and cantaloupe, suggesting the interaction between the Lcp and plant cellulose could be important in adhesion of *L. monocytogenes* (Bae et al., 2013).

Thus, the role of these cell surface elements in bacterial adhesion, flagella, fimbriae, O-antigens, type 3 secretion systems, and biofilm regulatory genes, remains unclear. Their role in adhesion to plants appears to depend on several factors, including the plant species under investigation, the plant organ, and the serotype/pathovar of bacteria being studied. Larger scale studies are urgently needed where a wide range of strains, genes and plant species are compared concurrently using the same experimental protocols to ensure experimental detail is not a factor. Further research can then address whether multiple mechanisms have evolved separately, whether there is specificity between mechanism and plant species or organ, or whether optimal attachment requires a combination of all three mechanisms together. A better understanding of these mechanisms could provide important targets for reducing the attachment of enteric pathogens to fresh produce.

### 3.2 Survival and colonization of enteric pathogens on fresh produce

Following adhesion to fresh produce, the ability of bacterial pathogens to survive and colonise produce surfaces is a key contributor to their ability to cause foodborne illness. Here, ‘survival’ is defined as the ability of the pathogen to survive on plant surfaces for extended periods of time, and ‘colonisation’ is the ability of the pathogen to multiply on the plant surface.

Microbial biofilms (see definition above) can form on leaves, fruit and root surfaces and within plant tissue, providing an adaptive strategy for bacteria to persist on plants, and resist disinfection treatments (reviewed in Yaron and Römling, 2014). As noted above, different bacterial strains can vary in their ability to form biofilms. Of particular note for the contamination of foods, *Salmonella* strains isolated from fresh produce formed stronger biofilms compared to those formed by *Salmonella* strains isolated from poultry (Patel et al., 2013). Furthermore, *Salmonella* strains that form stronger biofilms or produce greater quantities of biofilm adhere more strongly to leaf tissue (Kroupitski et al., 2009a,b; Patel and Sharma, 2010; Cevallos-Cevallos et al., 2012) compared to strains producing weak, or no biofilms. Similarly, *E. coli* isolated from plant hosts demonstrated significantly greater biofilm producing and extracellular matrix producing capabilities compared to isolates from mammalian hosts (Méric et al., 2013). Biofilm is also produced by *L. monocytogenes* on romaine lettuce leaves (Montgomery and Banerjee, 2015; Kyere et al., 2020). This suggests that biofilm formation may be an adaptive strategy for bacterial survival on plants.

Several studies provide consistent evidence that the pathogens can survive on leaves for periods ranging from several weeks to months. For example, *E. coli* O157:H7 and *S*. Typhimurium inoculated into compost could be detected from parsley leaves up to 177 and 231 days later, respectively, and from lettuce leaves up to 77 and 63 days later (Islam et al., 2004a; Islam et al., 2004b). These studies indicate potential differences in survival depending on the pathogen and plant species under investigation. However, lettuce sprayed with contaminated irrigation water containing *E. coli* O157:H7 resulted in recovery of the pathogen on leaves only up to 30 days post-spraying, although population numbers were not assessed after this time point, so it is possible that survival could occur over longer time periods (Solomon et al., 2003). After surface application of *L. monocytogenes* to different herbs, including basil, cilantro (coriander) and dill, the pathogen was detected for up to 28 days, although *L. monocytogenes* concentration was decreasing over time (Bardsley et al., 2019b).

Whilst many studies focus on the molecular mechanisms adopted by bacteria in initial attachment to fresh produce, less is understood about the genetic factors influencing survival on leaves or other plant surfaces, though as with initial attachment, flagella, biofilm components and fimbriae also appear to play a role. Johnson et al. (2020) have also demonstrated that *fliC*, as well as *sseB, hilD*, and *invA* (all involved in T3SS) are all required for survival of *S*. Typhimurium on lettuce. The biofilm formation gene, *ycfR*, is also involved in the survival of *E. coli* on lettuce roots (Hou et al., 2013). The role of biofilm components in survival may also differ depending on the location of the plant: colonic acid, which forms a protective capsule around bacterial cells, appeared to play a role in the survival of *E. coli* on lettuce leaves (Jang and Matthews 2018), but not on lettuce roots (Hou et al., 2013). The role of cellulose production in survival appears to differ between enteric species: whereas it was not involved in the survival of *E. coli* on lettuce (Jang and Matthews, 2018), it was implicated in the colonisation of sprouts with *S*. Newport (Barak et al., 2007). These results could indicate a greater role for cellulose production in both initial adhesion and survival on fresh produce by *Salmonella*, than in *E. coli* but would need further confirmation. Whilst there is little known about the genetic determinants influencing the colonisation of *L. monocytogenes* on plant leaves, evidence indicates root colonisation by *L. monocytogenes* is not mediated by prfA (biofilm/virulence factor), flagellin, or actA (virulence factor) (Schoenborn et al., 2021).

One aspect of colonisation is the ability of bacteria to multiply on the leaf surface. Two studies indicate a role for the T3SS in the colonisation of *Salmonella* on *Arabidopsis*, with *Salmonella* mutants deficient in SpvC, a T3SS effector protein, showing reduced population growth on leaves up to 96 h (Neumann et al., 2014). Similarly, genes involved in the structure of the *Salmonella* T3SS-1 and T3SS-2 are also involved in proliferation on *Arabidopsis* up to 72 h (Schikora et al., 2011). Both biofilm production and cellulose synthesis are involved in the proliferation of STEC on lettuce leaves (Fink et al., 2012). In addition to biofilm production and T3SS, iron acquisition was shown to be involved in survival of *Salmonella* on lettuce and alfalfa sprouts (Hao et al., 2012) and in proliferation on tomato fruit (Nugent et al., 2015). A comprehensive overview of the studies reporting colonisation and internalisation of *L. monocytogenes* highlights the molecular mechanisms involved in the interaction of the pathogen with plants, as well as the plant responses to the pathogen (Truong et al., 2021).

Given these wide differences in survival time across the different bacteria and plants, an understanding of pathogen population dynamics on plant surfaces is required. For this, the dynamics of other microbial communities on the plant surface should be considered, which could influence enteric pathogen survival, including beneficial, commensal, and other pathogenic microorganisms (Chialva et al., 2022). This has been demonstrated in *Arabidopsis*, where the phyllosphere microbiome elicited a protective effect against the fungal pathogen *Botrytis cinerea* (Ritpitakphong et al., 2016), and the bacterial pathogen *Pseudomonas syringae* (Vogel et al., 2021). Furthermore, comparisons of bacterial survival times across studies need to be considered with caution due to differences in inoculation methods and in initial titres of bacteria, as well as the resident phylloplane microbiota. Future experiments should compare all three enteric species across the same range of plant species of interest using a range of bacterial titres under varying environmental conditions and considering the phylloplane populations. Environmental factors which fluctuate under open-field conditions, such as humidity, temperature and solar radiation, may also need to be considered as they may affect bacterial survival. Whilst the impact of these variables on pathogen survival has been investigated under laboratory conditions, few studies have investigated their role under open-field conditions. For example, high relative humidity prior to harvesting tomatoes led to reduced *Salmonella* proliferation (Devleesschauwer et al., 2017). Similarly, solar radiation has been shown to directly impact the phyllosphere bacterial community on baby leaf lettuce (Truchado et al., 2017), and therefore could also impact colonisation by pathogens. Mathematic models have shown interesting correlations between *E. coli* and *S. enterica* colonisation of lettuce and spinach depending on weather stressors (Brandl et al., 2022). Moreover, genetic factors affecting growth and survival during the supply chain may be different following mechanical damage, and under chilled conditions with very different humidity to the field.

### 3.3 Enteric pathogens internalise through natural leaf openings

As well as colonising leaves, the ability of bacteria to internalise into plant tissue through natural openings on the surface enables them to avoid disinfection, which could provide one explanation as to why post-harvest processes may not be sufficient in reducing outbreaks. Stomatal pores present natural potential entry routes for enteric pathogens into leaves (reviewed in (Melotto et al., 2017), which may be preceded by stomatal colonisation. Indeed, several studies have observed colonisation around stomatal pores by *Salmonella* (Golberg et al., 2011; Yulia Kroupitski et al., 2009; Kroupitski et al., 2011; Kroupitski et al., 2019), *E. coli* (Itoh et al., 1998; Berger et al., 2009b; Shaw et al., 2008; Saldaña et al., 2011) and *L. monocytogenes* (Milillo et al., 2008; Mizan et al., 2020).

As with attachment and colonisation, a functional T3SS and flagella may be important for internalisation of *Salmonella* and *E. coli*. In STEC, tropism of bacteria towards stomata required a functional T3SS (Shaw et al., 2008; Saldaña et al., 2011), whereas in EAEC and *S*. Typhimurium, stomatal localisation was facilitated by flagella (Berger et al., 2009a, 2009b; Kroupitski et al., 2009a,b). Whilst stomatal colonisation does not necessarily indicate that the pathogens internalise through stomata, populations beneath the leaf surface of mutants lacking T3SS components and flagella were significantly reduced compared to wild type bacteria, indicating a role for these components in the internalisation of *S*. Typhimurium in lettuce (Johnson et al., 2020). This is further supported by Kroupitski et al. (2009a,b) who showed that disruption of flagella (*fliGHI*) and chemotaxis (*cheY*) gene expression led to reductions in the occurrence of *Salmonella* internalised within lettuce leaf tissue. As well as T3SS and flagella, several universal stress proteins (UspAB, YdaA, YecG and YbdQ) may also play a role in the internalisation of *S*. Typhimurium in lettuce, since mutants defective in these genes were not observed beneath the leaf surface, or within stomata (Kroupitski et al., 2019). Whilst much of the work to date has focussed on the internalisation of *E. coli* and *Salmonella* into plants (reviewed in Deering et al., 2012), less is understood about the genetic components mediating internalisation of *L. monocytogenes* into leaves despite the observation that it can internalise (Milillo et al., 2008; Shenoy et al., 2017), leaving an important knowledge gap to be filled.

## 4 Plants can respond to enteric pathogen presence, suggesting they are not passive vectors for transmission

As well as gaining an understanding of the bacterial mechanisms involved in contamination of fresh produce, understanding the role of the plant in these interactions is also critical. Whilst previously believed to be passive vectors for the transmission of enteric pathogens, a growing body of evidence indicates that enteric pathogens may in fact be recognised by plants. As sessile organisms, plants have evolved an innate immune system to detect and restrict plant pathogens, based on the recognition of bacterial cell surface molecules (Reviewed in Zipfel, 2014). This occurs through the recognition of cell surface pathogen-associated molecular patterns (PAMPs), or damage-associated molecular patterns (DAMPs), which are detected by cell surface localised pattern recognition receptors (PRRs) on the plant cells. This interaction between PAMPs and PRRs subsequently activates a downstream signalling cascade known as pathogen-triggered immunity (PTI), which confers resistance against a range of plant and human enteric pathogens. PTI involves several downstream processes including activation of mitogen-activated protein kinase (MAPK) genes, production of reactive oxygen species (ROS), enhanced expression of pathogenesis related (PR) genes, stomatal closure, and the activation of plant defence signalling pathways (Reviewed in Melotto et al., 2014). Whilst most work to date has focussed on the response of the plant defence system to plant pathogens, plant defence responses to enteric pathogens are receiving increasing attention. Interestingly, plant (*P. syringae*) and human (*E. coli* and *Salmonella*) pathogens appear to elicit both shared and unique mechanisms of the *Arabidopsis* defence response (Oblessuc et al., 2019; Oblessuc et al., 2020). Recognition of *S*. Typhimurium T3SS and flagella by salicylic acid (SA)- dependent and independent defence responses restrict *Salmonella* colonisation of *Arabidopsis thaliana* (Iniguez et al., 2005). Deciphering the genetic components influencing plant susceptibility to colonisation by human enteric pathogens, both in terms of the plant immune response and physical plant characteristics, could enable plant breeders to enhance food safety by producing varieties with reduced risk of contamination (reviewed in Henriquez et al., 2020; Melotto et al., 2020). Perhaps the most well-reported PAMP in enteric and plant pathogens is flagellin, recognised by the *Arabidopsis* FLS2 receptor, which detects a 22 amino acid region in the amino terminus of the flagellin protein (Flg22) (Chinchilla et al., 2006; Meng et al., 2013). Studies indicate that flagella-mediated PTI can be activated in *A. thaliana*, by both *S*. Typhimurium (Garcia et al., 2014) and *E. coli* (Seo and Matthews, 2012), as well as in *Nicotiana benthamiana* by *S*. Typhimurium (Meng et al., 2013). This recognition is highly specific, with the Flg22 epitope of *S*. Senftenberg resulting in a reduced ROS burst in tomato and *N. benthamiana* compared to the *S*. Typhimurium epitope, despite differing by only five amino acids (Garcia et al., 2014). However, plant immune responses to the flagellin epitope may be species specific, since a ROS burst is induced in tomato, but not in *N. benthamiana* or *Arabidopsis*, upon treatment with *E. coli* Flg22 epitopes (Robatzek et al., 2007). Whilst these studies indicate plant recognition of *S*. Typhimurium Flg22, *A. thaliana* leaves infiltrated with *S*. Senftenberg *fliC* flagellin mutants induced plant wilting, indicating Flg22 perception may not be responsible for the leaf wilting response (Berger et al., 2011). Interestingly, it has been shown that *Salmonella* may express flagellin (*fliC* vs *fljB*) heterogeneously across a population when in contact with tomato leaves (Zarkani et al., 2020), and this may act to evade host response. Whilst limited work has been performed on the activation of plant defence by *L. monocytogenes*, it has been shown that *A. thaliana* does not respond to the flagella of *L. monocytogenes*, as the growth of wild-type compared to *flaA* mutants was not significantly different on *Arabidopsis* roots, nor was there an induction of MAPK gene expression following inoculation with *L. monocytogenes* (Truong et al., 2021).

Other PAMPs present on *Salmonella* cell surfaces could also elicit PTI. This is supported by Garcia et al. (2014), who showed that there was still some induction of PTI marker genes in *Arabidopsis* FLS2 mutants following inoculation with *S*. Typhimurium. LPS is another PAMP and indeed, purified LPS from *S*. Typhimurium induced a ROS burst in *N. tabacum* (Shirron and Yaron, 2011). However, recognition of LPS may also be plant species-specific as no response was observed in tomato (Meng et al., 2013). The role of LPS as a PAMP is further supported by Berger et al. (2011), who demonstrated a range of *S*. Senftenberg strains from serogroup E(4), which possess O antigen 1,3,19, induced leaf chlorosis and wilting in *Arabidopsis*, unlike strains lacking the O antigens, suggesting that the O antigen part of the LPS may be recognised by the plant. Interestingly, LPS in STEC may play a role in suppressing the plant immune system since STEC mutants with truncated LPS elicited increased Pathogenesis Related 1 (*PR1*) gene expression 8 h post-inoculation in *Arabidopsis* (Jang and Matthews, 2018). Whereas the PRR involved in plant pathogenic LPS recognition was identified as a lectin S-domain receptor kinase in *Arabidopsis* (Ranf et al., 2015), this receptor seems unable to detect LPS from *S*. Typhimurium or *E. coli* and it is as yet unknown if it is responsible for the detection of *S*. Senftenberg. The identification of PRR’s in plants is usually discussed in terms of engineering resistance against plant pathogens, but it could also enhance plant resistance against colonisation by enteric pathogens, potentially reducing foodborne illness outbreaks.

Differences in plant genotype also play a role in defence responses to enteric pathogens. When infiltrated into different lettuce cultivars, both *S*. Typhimurium and *E. coli* elicited a greater ROS burst in the ‘lollo rossa’ lettuce cultivar compared to the ‘red tide’ cultivar (Jacob and Melotto, 2020), suggesting a greater defence response elicited by ’lollo rossa’. Plants may also respond differently to different bacterial species, indicating species-specific induction of plant immune responses. STEC induced greater expression of PR1 genes than did *S*. Typhimurium in both *Arabidopsis* and lettuce (Roy et al., 2013), while ethylene and jasmonic acid signalling genes were induced more strongly by *S*. Typhimurium relative to STEC in *Medicago truncatula* (Jayaraman et al., 2014). These results may be explained by the ability of different enteric pathogens to suppress plant immune responses. Similarly, different pathogen serovars can elicit differential induction of plant defences: Jang and Matthews (2018) showed that there was reduced *PR1* gene induction in *Arabidopsis* inoculated with *E. coli* O104:H4, a better coloniser of plants, relative to O157:H7. This was hypothesised to be caused by higher amounts of capsular polysaccharide on O104:H4, which could mitigate the host response and thus increase survival on plants. Whilst human bacterial pathogens are not associated with physical disease symptoms of plants, studies reviewed here indicate that human enteric pathogens can be recognised by them. However, several questions remain to be addressed, including whether enteric pathogen detection by plants is a consequence of sequence similarity to highly conserved motifs in plant pathogens, or whether they are recognised by plants due to their ability to cause potential harm.

One defence response elicited by the presence of PAMPs is stomatal closure, mediated by FLS2 recognition of flagellin (Melotto et al., 2006). As stomata may act as entry routes for plant pathogen invasion, plants have developed strategies to close them following pathogen recognition, preventing entry and pathogenesis (reviewed in Melotto et al., 2017). However, some plant pathogens have developed different mechanisms to inhibit stomatal closure. Similarly, certain human enteric pathogens may also have evolved similar strategies. Whilst inoculation of lettuce with *E. coli* (Roy et al., 2013) or *L. monocytogenes* (Johnson et al., 2020) led to long-term stomatal closure, *S*. Typhimurium inoculation caused only temporary closure in both studies, suggesting the pathogen may have developed strategies to overcome long term stomatal closure. Whereas several components of the T3SS-2, including SseB (T3SS-2) and HilD (T3SS SPI1 and 2), are involved in preventing stomatal closure (Johnson et al., 2020), T3SS-1 does not affect the stomatal closure (Shirron and Yaron, 2011), despite its involvement in the suppression of a ROS burst in tobacco. The ability of *S*. Typhimurium to prevent stomatal closure has been observed in both ‘romaine’ and ‘butterhead’ lettuce inoculated with *S*. Typhimurium, but plant species and environmental conditions, including temperature and humidity, impact the ability of *S*. Typhimurium to prevent stomatal closure (Roy and Melotto, 2019). These findings indicate the possibility of plant species-specific adaptation by *S*. Typhimurium, which may also be influenced by a range of environmental factors, and highlights the importance of storage temperatures in reducing pathogen internalisation.

## 5 Physical plant characteristics can influence bacterial contamination

Physical and biochemical plant surface characteristics can also act as a defence to bacterial pathogens, and therefore may also play a role in levels of contamination. Leaf surfaces vary in their macromorphology, including veins and margins, in their micromorphology, including stomatal size and density, and the presence of trichomes, which are appendages on plant surfaces often involved in the biosynthesis of defence compounds. The surface properties of leaves also vary in their hydrophobicity, dependent on epicuticular waxes and hydathodes, which are pores that exude water onto the leaf surface. Leaf age also influences leaf surface properties (Busta et al., 2017). Leaf age has been shown to affect adhesion, although results are contradictory. Brandl and Amundson (2008) observed greater levels of *Salmonella* and *E. coli* on younger compared to older leaves, whereas other studies found that older lettuce leaves supported higher adhesion levels of *Salmonella* than younger leaves (Kroupitski et al., 2011; Hunter et al., 2015). However, differences in the ages of lettuce leaves tested across the studies, as well as inoculation conditions, make it difficult to compare findings. Macromorphology has been shown to influence levels of attachment, whereby *S*. Typhimurium appeared to preferentially attach closer to the petiole than the leaf blade, and greater attachment was observed on rougher areas of the leaf (Kroupitski et al., 2011). Similarly, *S*. Thompson has been observed at specific sites of the leaf, including the veins of cilantro leaves (Brandl and Mandrell, 2002). Contrastingly, *S*. Senftenberg showed more evenly distributed adhesion patterns on the surface of leaves, without association to the typical bacterial adhesion sites, for both lettuce (Hunter et al., 2015) and basil (Berger et al., 2009a). Several studies have shown that *S*. Typhimurium has specific colonisation sites, on both lettuce (Jechalke et al., 2019), and tomato leaves (Gu et al., 2013) including hydathodes, and the adhesion patterns of *Salmonella* appear to be serovar specific, though whether more hydathodes result in higher internalisation is not known. Whilst fewer studies have been performed on the leaf attachment mechanisms of *L. monocytogenes*, Gorksi and colleagues showed preferential binding to the veins of lettuce (Gorski et al., 2021). At a micromorphological level, higher rates of internalisation of *E. coli* and *Salmonella* on lettuce were associated with greater stomatal width and area, although stomatal density did not significantly correlate with internalisation rates (Jacob and Melotto, 2020). Type I trichomes have also been identified as sites of adhesion for *Salmonella* on tomato leaves (Barak et al., 2011; Cevallos-Cevallos et al., 2012), *E. coli* on lettuce leaves (Brandl and Amundson, 2008), and all three pathogens reviewed here on peach fruit (Collignon and Korsten, 2010). As well as the physical characteristics affecting levels of adhesion, leaf metabolites have shown a role in the colonisation of *Salmonella* across different tomato cultivars, with higher amounts of sugars, sugar alcohols and organic acids being correlated with increased *S. enterica* growth (Han and Micallef, 2014).

## 6 Conclusions and future perspectives

Statistics from the United Nations indicate that by 2100, the global population will increase to around 10.4 billion people (United Nations Department of Economic and Social Affairs, 2022). To provide sufficient food for the growing population, increasing crop yields is fundamental, although reducing foodborne-illness outbreaks and subsequent waste from product recalls can also contribute to achieving food security. Furthermore, as urban populations expand into the countryside and disposal of refuse and human waste becomes an increasing problem, the risks of contaminating crops with human enteric pathogens could increase. Understanding plant-microbe interactions with a view to reducing attachment and colonisation throughout the supply chain could be an important contribution to mitigating this increased risk.

Reducing foodborne illness outbreaks needs to consider both pre- and post-harvest practices. This would be included in a “One Health” approach that takes into account sustainable farming production, maintaining a healthy ecosystem in horticultural production, as well as ensuring the safety of the produce for human consumption (Yan et al., 2022). Hazard analysis at the crop production level needs to be supported by more scientific-based evidence, including, for example, which irrigation systems are better or worse for supporting bacterial contamination, and where irrigation systems are placed in relation to livestock grazing. This has been highlighted by the FDA, who suggests a combined effort across growers, ranchers, and federal agencies is needed to prevent further outbreaks caused by livestock grazing (FDA, 2022). Good manufacturing practice during post-harvest processing, including sufficient sanitisation of machines, is also critical due to the ability of pathogens to persist on surfaces (Gil et al., 2015), although more research is needed to understand the mechanisms of attachment and whether they differ across enteric species. Vertical farming, which involves growing commercial crops in stacked layers under controlled environmental conditions, is receiving growing interest due to the potential to increase crop yields per unit area of land. The bacterial community structure on leaves from vertically grown rocket salad differed from that of other farming methods (Mantegazza et al., 2023). This suggests that pest and disease management of crops within these systems, both in terms of crop pests and pathogens (Roberts et al., 2020), but also human pathogens, is an area of future research priority.

Whilst microbial biocontrol agents are receiving increasing attention for their use in agricultural productivity, there is evidence that they could also be harnessed for food safety applications, to reduce attachment and colonisation of human enteric pathogens through a form of biological control. Current research areas of interest include the use of bacteriophages (viruses specific to bacteria). This approach has been shown as promising to control agents of enteric, foodborne pathogens (Reviewed in Kazi and Annapure, 2016). One example is SalmoFresh™, containing lytic bacteriophages specific to several *Salmonella* serovars, which successfully reduced populations on lettuce and cantaloupe rinds (Zhang et al., 2019). This is a promising new avenue for the reduction of pathogen contamination on fresh produce crops. As well as bacteriophages, beneficial soil microbes may also offer potential for reducing enteric pathogen populations. Soil microbial inoculants containing a consortium of biocontrol agents demonstrated efficacy at reducing growth of *L. monocytogenes*, which may in part be explained by the production of inhibitory secondary metabolites (Sharma et al., 2020). Similarly, when applied to seeds of spinach, beneficial *Pseudomonas* species showed a reduction in *E. coli* populations under field trials (Uhlig et al., 2021), highlighting a promising approach for reducing future foodborne illness outbreaks. Protective cultures can also be used to reduce enteric pathogen contamination of fresh fruits and vegetables, including lactic acid bacteria, which produce bacteriocins possessing antimicrobial activity against foodborne pathogens (Agriopoulou et al., 2020).

Whilst progress has been made in developing an understanding of the molecular mechanisms involved in bacterial adhesion to plants, several priority areas for future research emerge. Many studies target specific bacterial adhesins, and whilst deletion of certain components results in log fold reductions in bacterial counts, they do not eliminate them, suggesting multiple cell surface appendages are involved in adhesion, which may also act synergistically. Although *Salmonella* and *E. coli* are well studied for their adhesion to fresh produce, *L. monocytogenes* has received far less attention, although appendages from *L. monocytogenes* have been observed on the surface of spinach leaves (Fig. 4B). As seeds have also been shown to be an important source of contamination, understanding the molecular components, and therefore mechanisms, involved in bacterial attachment to seeds is also critical, and indeed developing effective decontamination treatments. Further work is also required to better understand mechanisms of attachment to fruit surfaces. Moreover, particularly with ready-to-eat mixed-bagged salads, future efforts should determine whether particular species or cultivars of leaves found in mixed salads are more or less supportive of human enteric pathogen growth. This would enable the selection of cultivars or species less supportive of pathogens, to improve food safety. These aspects of food safety are receiving increasing attention (Henriquez et al., 2020; Melotto et al., 2020). More rapid, cost-effective and easy-to-use methods for detection of contamination through the supply chain would also be beneficial for early detection and removal of contaminated material before it reaches the consumer. The use of volatile organic compounds (VOCs) shows promise of a new approach for this. Changes in VOC patterns have been used successfully to detect *L. monocytogenes* on cantaloupe (Spadafora et al., 2016), and could be used alongside molecular detection techniques through e.g. PCR and established, but slow, culturing methods (reviewed in Lee et al., 2015). Further research, addressing mechanisms driving the associations between enteric pathogens and plants and how to mitigate and detect contamination, is required to provide evidence-based policies to reduce foodborne illness outbreaks.

## Figures and Tables

**Table 1:** Number of outbreaks, case numbers and deaths associated with contaminated fruit/vegetables between 2006 and 2023 in the USA. (Data from The Centers for Disease Control, July 12, 2023).

Sources	*Salmonella*				*E. coli*				*L. monocytogenes*		
	Outbreaks	Cases	Deaths		Outbreaks	Cases	Deaths		Outbreaks	Cases	Deaths
Tomatoes	1	183	0		0	0	0		0	0	0
Sprouted vegetables	9	694	0		4	110	0		1	5	2
Papayas	6	438	3		0	0	0		0	0	0
Melon/Cantaloupe	5	546	0		0	0	0		1	147	33
Coconut	2	41	0		0	0	0		0	0	0
Cucumbers	3	1266	7		0	0	0		0	0	0
Mangoes	1	127	0		0	0	0		0	0	0
Spinach	0	0	0		3	247	3		0	0	0
Lettuce	0	0	0		5	523	5		0	0	0
Mixed fruit/vegetables/salads	3	1638	0		4	118	2		5	75	8
Apples	0	0	0		0	0	0		1	35	7
Mushrooms	1	55	0		0	0	0		2	41	4
Onions	2	2167	0		0	0	0		0	0	0
Peaches	1	101	0		0	0	0		0	0	0
**Total**	**34**	**7256**	**10**		**16**	**998**	**10**		**10**	**303**	**54**

**Table 2:** Role of enteric bacterial genes in adhesion to fresh produce.

Function	Gene	Organisms	Plant species*	Plant organ	Role in attachment	Reference
**Flagella related**	*fliC*	STEC	Spinach (*Spinacia oleracea*)	Leaves	Yes	Saldaña et al., 2011
		EHEC	Spinach/Lettuce	Leaves	Yes	Xicohtencatl-Cortes et al. (2009); Nagy et al. (2015)
		ETEC	Rocket (*Eruca vesicaria*)	Leaves	Yes	Shaw et al. (2011a)
		EAEC	Rocket (*E. vesicaria*)	Leaves	No	Berger et al. (2009b)
		*S*. Typhimurium	Basil	Leaves	No	Berger et al. (2009a)
		*S*. Senftenberg	Basil	Leaves	Yes	Berger et al. (2009a)
	*fliC/fljB*	*S*. Senftenberg	Tomato (*Solanum lycopersicum*)	Fruit	No	Shaw et al. (2011b)
		*S*. Typhimurium	Corn salad (*Valerianella locusta*)	Leaves	Yes	Elpers et al. (2020)
	*fliGHI*	*S*. Typhimurium	Lettuce (*Lactuca sativa*)	Leaves	Yes	Kroupitski et al. (2009a,b)
	*motAB*	*S*. Typhimurium	Corn salad (*V. locusta*)	Leaves	Yes	Elpers et al. (2020)
	*cheY/cheZ*	*S*. Typhimurium	Corn salad (*V. locusta*)	Leaves	No	Elpers et al. (2020)
	*flaA*	*L. monocytogenes*	Alfalfa/Radish/Broccoli	Sprouts	Variable	Gorski et al. (2009)
	*motAB*	*L. monocytogenes*	Alfalfa/Radish/Broccoli	Sprouts	No	Gorski et al. (2009)
**Fimbriae**	*csgA*	STEC	Lettuce (*L. sativa*)	Leaves	Yes	Fink et al. (2012)
		STEC	Spinach (*S. oleracea*)	Leaves	Yes	Macarisin et al. (2012); Saldaña et al., 2011; Carter et al. (2016)
		STEC	Alfalfa	Sprouds/seed coats	No	Torres et al. (2005)
	*escN*	ETEC	Rocket (*E. vesicaria*)	Leaves	Yes	Shaw et al. (2008)
		EHEC	Spinach	Leaves	Yes	Xicohtencatl-Cortes et al. (2009)
		STEC	Spinach (*S. oleracea*)	Leaves	Yes	Saldaña et al., 2011
	*aaf*	EAEC	Rocket (*E. vesicaria*)	Leaves	Yes	Berger et al. (2009b)
	*aag*	EAEC/STEC	Spinach/Lettuce	Leaves	No	Nagy et al. (2015)
	*agfA*	*S*. Enteritidis	Alfalfa	Sprouts	No	Barak et al. (2005)
	*agfB*	*S*. Enteritidis	Alfalfa	Sprouts	Yes	Barak et al. (2005)
	*rpoS*	*S*. Newport	Alfalfa	Sprouts	Yes	Barak et al. (2005)
***O-antigen capsule***	*rfbE*	STEC	Lettuce	Leaves	Yes	Boyer et al. (2011)
	per	STEC	Spinach	Leaves	No	Nagy et al. (2015)
	*waal*	STEC	Alfalfa	Sprouts	No	Matthysse et al. (2008)
	*yihO*	*S*. Enteritidis	Alfalfa	Sprouts	Yes	Barak et al. (2007)
	*wzz*	*S*. Typhimurium	Corn salad (*V. locusta*)	Leaves	Yes	Elpers et al. (2020)
	*fepE*	*S*. Typhimurium	Corn salad (*V. locusta*)	Leaves	No	Elpers et al. (2020)
	*rfaL*	*S*. Typhimurium	Corn salad (*V. locusta*)	Leaves	No	Elpers et al. (2020)
	*wzz/fepE*	*S*. Typhimurium	Corn salad (*V. locusta*)	Leaves	Yes	Elpers et al. (2020)
**Biofilm**	*sab*	STEC	Rocket (*E. sativa*)	Leaves	Yes	Abe et al. (2020)
	*flu*	STEC	Rocket (*E. sativa*)	Leaves	Yes	Abe et al. (2020)
	*ycfR*	*E. coli* K-12	Lettuce (*L. sativa*)	Leaves	No	Fink et al. (2012)
		*S*. Typhimurium/Saintpaul	Spinach/grape tomato	Leaves/fruit	Yes	Salazar et al., 2013a,b
		*S*. Typhimurium	Cabbage	Leaves	No	Kim and Yoon (2019)
	*sirA*	*S*. Typhimurium/ Saintpaul	Spinach/grape tomato	Leaves/fruit	Yes	Salazar et al., 2013a,b
	*bapABCD*	*S*. Typhimurium	Corn salad (*V. locusta*)	Leaves	Yes	Elpers et al. (2020)
	*lmo0753*	*L. monocytogenes*	Lettuce/Cantaloupe	Leaves/Fruit	Yes	Salazar et al., 2013a,b
***Cellulose binding genes***	*bcsA*	STEC	Spinach (*Spinacia oleracea*)	Leaves	No	Macarisin et al. (2012); Saldaña et al., 2011
		*S*. Enteritidis	Alfalfa	Sprouts	Yes	Barak et al. (2007)
		*S*. Typhimurium	Plant cell wall models	N/A	Yes (Temperature dependent)	Tan et al. (2016)
	*bcsB*	STEC	Alfalfa	Sprouts	Yes	Matthysse et al. (2008)
		*S*. Typhimurium	Parsley (*Petroselinum crispum*)	Leaves	Yes	Lapidot and Yaron (2009)
	*bcsC*	*S*. Typhimurium	Tomato (*S. lycopersicum*)	Fruit	Yes	Shaw et al. (2011b)
	*csgD*	STEC	Spinach (*S. oleracea*)	Leaves	Yes	Saldaña et al., 2011
		*S*. Typhimurium	Plant cell wall models	N/A	No	Tan et al. (2016)
	lep	*L. monocytogenes*	Lettuce/spinach/	Leaves/fruit skin	Yes	Bae et al. (2013)
	protein		cantaloupe			

* Latin names given in parentheses if stated in paper

## Data Availability

No data was used for the research described in the article.
